# Antibacterial properties of *Buddleja globosa*: a review of extracts and bioactive compounds

**DOI:** 10.3389/fphar.2026.1896014

**Published:** 2026-08-18

**Authors:** Carolina V. Delgado-Gazzoni, Pamela A. Pérez-Basáez, Maria V. Bruna, Henry E. Garrido-Vera, Caroline R. Weinstein-Oppenheimer, Tania F. Bahamondez-Canas

**Affiliations:** 1 Escuela de Química y Farmacia, Facultad de Farmacia, Universidad de Valparaíso, Valparaíso, Chile; 2 Centro de Investigación, Desarrollo e Innovación en Productos Bioactivos (CInBIO), Universidad de Valparaíso, Valparaíso, Chile

**Keywords:** antibacterial activity, antibiofilm, antibiotics, *Buddleja globosa*, synergism

## Abstract

*Buddleja globosa* (Buddlejaceae) is a South American medicinal plant traditionally used for wound healing and other inflammatory conditions. In recent years, increasing attention has been given to its antibacterial potential and to the bioactive compounds that may contribute to this activity. This review summarizes the available evidence on the antibacterial-related activity of *B. globosa* extracts and selected metabolites reported in the species, including direct antibacterial effects, antibiofilm activity, antivirulence mechanisms, host-protective effects, and antibiotic-adjuvant properties. The available evidence indicates that polar extracts of *B. globosa*, particularly aqueous, hydroalcoholic, and ethanolic preparations, can inhibit clinically relevant bacteria such as *Staphylococcus aureus, Escherichia coli*, and *Pseudomonas aeruginosa*, although reported activity varies according to extract type, plant material, and assay methodology. Among the identified metabolites, phenylethanoid glycosides, especially verbascoside and forsythoside B, are the most relevant compounds due to their abundance in *B. globosa* extracts and their reported antibacterial-related mechanisms. Verbascoside shows variable direct antibacterial activity but stronger evidence as an antibiofilm, antivirulence, antitoxin, and host-protective compound. Forsythoside B displays direct activity against selected Gram-negative pathogens and potentiates conventional antibiotics against multidrug-resistant bacteria. Flavonoids such as luteolin, apigenin, quercetin, and their glycosides also contribute relevant antibacterial, antibiofilm, or antivirulence evidence, although many studies have used commercial standards or compounds isolated from other plant species. Overall, *B. globosa* represents a promising source of antibacterial-related bioactive compounds, but future studies should prioritize standardized extract characterization, quantitative susceptibility testing, cytotoxicity assessment, mechanistic validation, and *in vivo* models that connect phytochemical composition with antibacterial efficacy.

## 1 Introduction

### 1.1 Antibacterial resistance, biofilms, and the need for complementary therapeutic strategies

Antibacterial resistance remains one of the most important challenges in infectious disease treatment. The emergence and dissemination of multidrug-resistant bacteria have reduced the effectiveness of conventional antibiotics and limited therapeutic options for several clinically relevant pathogens, including *Acinetobacter baumannii, Pseudomonas aeruginosa, Staphylococcus aureus, Streptococcus pneumoniae, Klebsiella pneumoniae*, and *Escherichia coli* (WHO, 2020; Reygaert, 2018). Bacterial resistance can arise through multiple mechanisms, including enzymatic inactivation of antibiotics, reduced membrane permeability, active efflux systems, and modifications of antibiotic targets. These mechanisms are particularly concerning in pathogens associated with hospital-acquired infections, chronic wounds, respiratory infections, and urinary tract infections, where treatment failure can increase morbidity, healthcare costs, and the risk of recurrent infection (Percival et al., 2015a; Maugeri et al., 2019).

In addition to classical resistance mechanisms, bacterial biofilms represent a major barrier to effective antibacterial therapy. Biofilms are structured microbial communities embedded in an extracellular polymeric matrix composed mainly of polysaccharides, proteins, extracellular DNA, and glycoproteins (Bjarnsholt, 2013). This matrix protects bacteria from antibiotics and host immune responses, while also favoring persistence, recurrence, and dissemination of infection (Percival et al., 2015b). Therefore, antibacterial research increasingly considers not only direct growth inhibition, measured by minimum inhibitory concentration (MIC), but also antibiofilm activity, inhibition of adhesion, disruption of virulence mechanisms, toxin neutralization, and antibiotic potentiation. These antibacterial-related mechanisms are especially relevant because they may reduce bacterial pathogenicity or restore antibiotic susceptibility without necessarily acting as conventional bactericidal agents.

Despite the increasing clinical impact of antibacterial resistance, the development of new antibacterial agents has remained limited during recent decades. Only a small number of new antibacterial classes have reached clinical practice, while many current treatments still rely on older antibiotic families whose efficacy is increasingly compromised by resistance mechanisms (Cole, 2014; Wagenlehner et al., 2024; Lewis, 2012). This limited innovation pipeline has renewed interest in complementary strategies, including compounds that can restore antibiotic susceptibility, inhibit biofilm formation, attenuate bacterial virulence, or enhance host protection during infection. In this context, plant-derived metabolites represent a relevant source of chemically diverse molecules with potential antibacterial-related activity.

Natural products are particularly relevant in this context because plants produce chemically diverse secondary metabolites involved in defense against microbial colonization and environmental stress (Scur et al., 2016). These compounds can act through multiple mechanisms, including membrane disruption, interference with bacterial metabolism, inhibition of biofilm formation, modulation of virulence factors, and synergistic interactions with antibiotics (Bibow and Oleszek, 2024). Thus, plant-derived compounds may contribute to the development of antibacterial adjuvants, antivirulence agents, or topical anti-infective formulations.

### 1.2 *Buddleja globosa* as a source of antibacterial-related bioactive compounds

**FIGURE 1:**
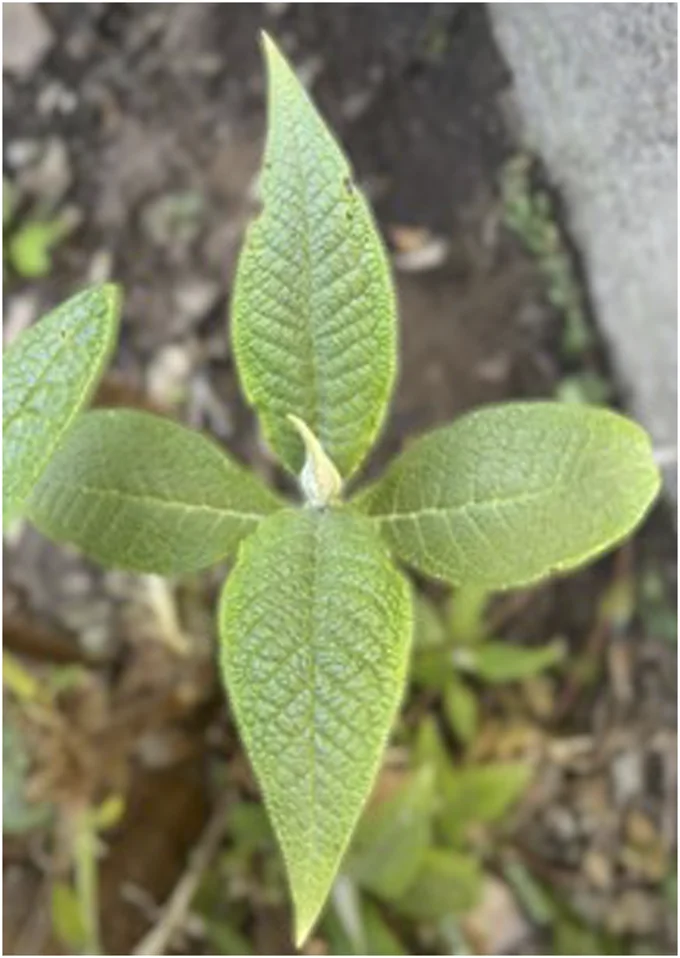
Leaves and apical meristem of *Buddleja globosa* Hope. Opposite leaves showing the characteristic lanceolate shape, serrate margins, and prominent reticulate venation (Pamela Pérez-Basáez).

*Buddleja globosa*, commonly known as matico, is an evergreen shrub native to South America, particularly Chile, Peru, and Argentina that usually reaches 1.5–3 m in height, with yellowish semi-woody stems and opposite, ovate-lanceolate leaves that are rugose, acute at the apex, and whitish on the abaxial surface (Figure 1). Its flowers are arranged in distinctive globose heads, typically yellow to orange or reddish, while the fruit is a small capsule containing numerous polyhedral seeds. This botanical characterization is relevant because the leaves and aerial parts are the plant materials most frequently used in traditional preparations and phytochemical studies. It has a long history of traditional use, especially for wound healing and the treatment of inflammatory conditions, and is commonly prepared as infusions, poultices, or topical applications from leaves and stems (Backhouse et al., 2008; Houghton, 1984; Mensah et al., 2001). These traditional uses have encouraged pharmacological studies on its antioxidant, anti-inflammatory, wound-healing, and antibacterial properties.

The phytochemical profile of *B. globosa* is dominated by phenylethanoid glycosides, flavonoids, phenolic acids, and terpenoid constituents (Torres-Vega et al., 2021). Among these, verbascoside, also known as acteoside, has been consistently reported as one of the major marker compounds of *B. globosa* leaves. Other relevant compounds include isoverbascoside, forsythoside B, luteolin-7-O-glucoside, apigenin-7-O-glucoside, quercetin derivatives, caffeoyl derivatives, catechin, and selected terpenoid compounds. However, the chemical composition of *B. globosa* extracts can vary according to plant organ, leaf maturity, seasonal variation, drying conditions, and extraction method (Vogel et al., 2002; Hevia et al., 2006). This variability is important when interpreting antibacterial data, because differences in phytochemical composition may partly explain differences in reported biological activity. Importantly, this review distinguishes between compounds that have been chemically identified in *B. globosa* and the sources from which the corresponding biological evidence was obtained. In many cases, the antibacterial or antibacterial-related activities discussed here were evaluated using commercially available standards or the same compounds isolated from other plant species, rather than from compounds purified directly from *B. globosa*. These studies remain relevant for understanding the potential pharmacological contribution of metabolites present in *B. globosa*, but they should be interpreted as indirect evidence of the biological relevance of the species rather than as direct demonstrations of activity from *B. globosa*-derived purified compounds. Supplementary Table S1 shows the compounds isolated from *B. globosa* extracts separated by their corresponding classification.

Several studies have evaluated the antibacterial activity of *B. globosa* extracts and selected compounds associated with the species. However, the available evidence is heterogeneous. Some studies evaluate crude aqueous, hydroalcoholic, ethanolic, methanolic, or essential oil extracts, whereas others investigate isolated compounds, commercial standards, or metabolites obtained from other botanical sources but also reported in *B. globosa*. In addition, antibacterial activity has been assessed using different methods, including agar diffusion, broth dilution, biofilm assays, cell infection models, and *in vivo* infection models. Therefore, a critical synthesis is needed to distinguish direct antibacterial effects from broader antibacterial-related mechanisms, such as antibiofilm activity, antivirulence effects, host-cell protection, and antibiotic-adjuvant activity.

This review summarizes the current evidence on the antibacterial-related properties of *B. globosa* extracts and selected compounds identified in the species. Particular emphasis is placed on clinically relevant bacterial pathogens, biofilm-associated activity, mechanisms of bacterial inhibition or virulence attenuation, and synergistic or potentiating interactions with conventional antibiotics. By organizing the evidence according to extract type, metabolite relevance, and mechanism of action, this review aims to clarify the antibacterial potential of *B. globosa* and identify the main gaps that should be addressed in future pharmacological and translational studies.

## 2 Methodology

The literature search was conducted in two stages. In the first stage, the PubMed database was searched using the terms “Buddleja globosa AND antimicrobial”, “Buddleja globosa AND antibacterial”, and “Buddleja globosa AND biofilm”. The Boolean operator AND was used in all searches. This search identified 69 records. After removing 15 duplicate records, 54 articles remained for screening. Exclusion criteria included review articles, studies evaluating metabolites exclusively as part of formulations, semi-synthetic derivatives, studies not focused on microorganisms, and studies unrelated to human health. Following the screening process, 6 articles met the eligibility criteria and were included in the review.

**FIGURE 2:**
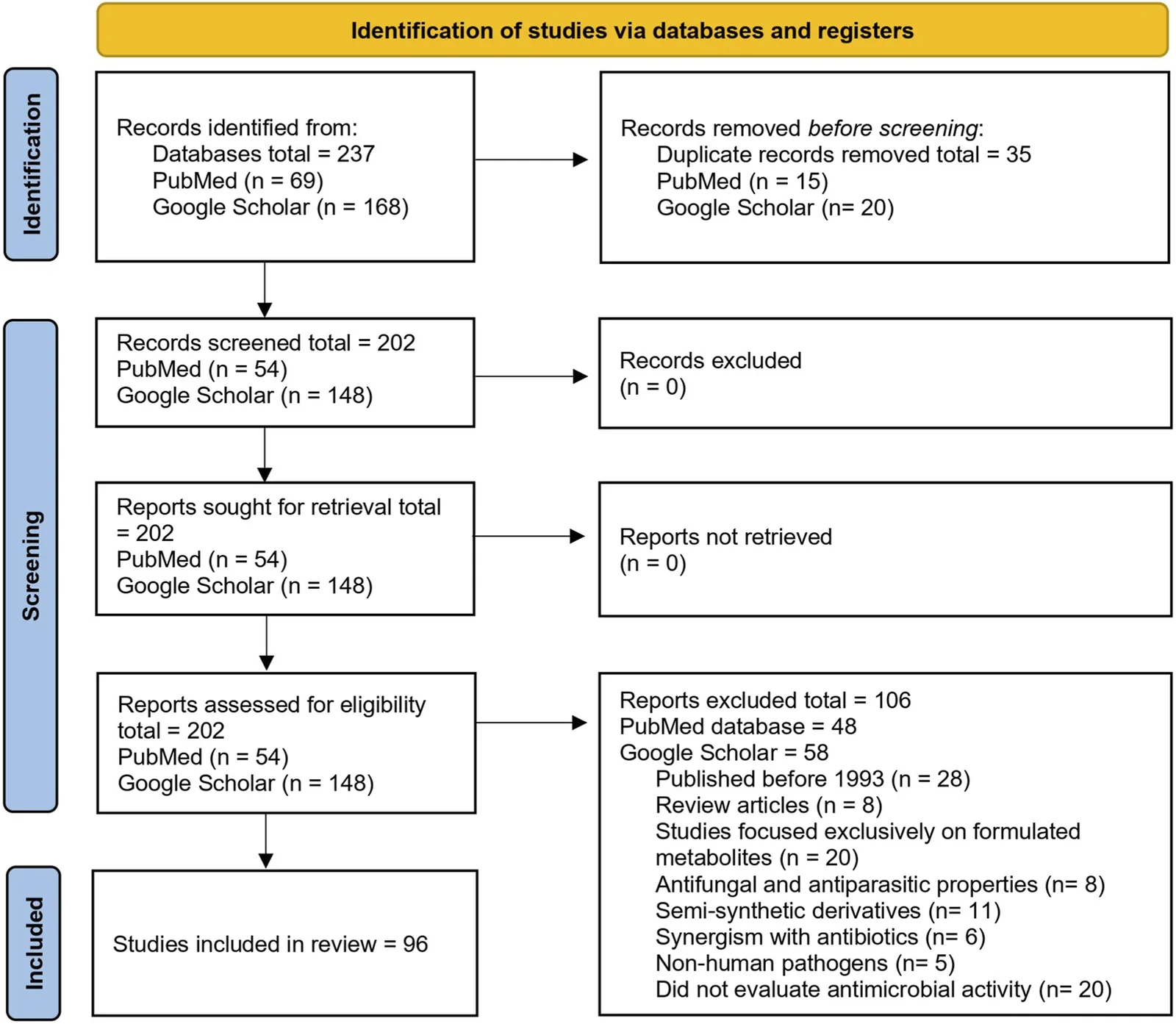
Flow diagram illustrating the literature search and study selection process that informed this review.

In the second stage, the literature search was updated in Google Scholar to identify additional evidence regarding the metabolites previously identified in *B. globosa*. Each relevant metabolite was combined with the terms “antimicrobial”, “antibacterial”, and “biofilm”, and additional searches were performed using the terms “Buddleja globosa AND antimicrobial”, “Buddleja globosa AND antibacterial”, and “Buddleja globosa AND biofilm”. The Boolean operator AND was used in all searches. This updated search identified 168 records. After removing 20 duplicate records, 148 articles remained for screening. The same eligibility criteria applied during the first stage were used, resulting in the inclusion of 90 additional articles. Overall, 96 articles met the eligibility criteria and were included in this review. Figure 2 shows the literature search and study selection process used in this review.

## 3 Results

### 3.1 Scientific evidence of the antibacterial properties of *Buddleja globosa* extracts

#### 3.1.1 Aqueous extracts

Among the first studies, Pérez and Anesini evaluated the activity of various extracts from medicinal plants native to Argentina, including *B. globosa*. They used the aerial parts of the plant, previously air-dried, and then made an infusion. This was evaluated by the agar plate diffusion method, on *Salmonella enterica* Typhimurium. The researchers compared the inhibition zone generated by the extract after 48 h to ampicillin, but the extract did not show antimicrobial activity on this species (Pérez and Anesini, 1994). On the other hand, Hermosilla et al., developed aqueous *B. globosa* leaf extracts with an MIC against *S. aureus* at 0.25 mg/mL (Hermosilla et al., 2026).

#### 3.1.2 Organic extracts

Pardo et al. determined the bacteriostatic activity of an ethanolic extract of dry leaves of *B. globosa* on strains of *S. aureus* and *E. coli* (Pardo et al., 1993). Hydroethanolic extracts of *B. globosa* leaves have shown an MIC of 0.25–0.5 mg/mL against *S. aureus* (Mensah et al., 2000). On the other hand, the methanolic extracts of leaves and stems of *B. globosa* evaluated by Mølgaard et al. did not show activity on the bacterial species tested (Mølgaard et al., 2011). The authors used leaves and stems collected during the summer that were dried at 40 °C for 72 h prior to extraction. The extracts were evaluated on *Bacillus subtilis, P. aeruginosa, S. aureus*, and *E*. *coli*, but there was no activity at a maximum amount of 1 mg of each extract.

Pellegrini et al. were the first authors to characterize an essential oil of *B. globosa*, finding bioactive compounds that had not been previously identified (Pellegrini et al., 2017). The oil showed good antibacterial activity against *Paenibacillus larvae* (a bacteria that affects honeybees), with a minimum inhibitory concentration (MIC) of 25 μg/mL. Although this oil was evaluated on a microorganism that is not pathogenic for humans, the extract could affect the membrane of *P. larvae*, allowing greater permeability of the crystal violet solution, along with the loss of cellular material. Therefore, the study would contribute to the identification of the mechanism of action of the extract. Table 1 summarizes *B. globosa* extracts that have been evaluated in microbial species.

**TABLE 1:** Antibacterial properties of *Buddleja globosa* extracts studied *in vitro*.

Extract	Organ	Method	Range	Species	Ref.
Aqueous	Leaves	Agar diffusion	ND	*S. typhi*	Pérez and Anesini (1994)
		Broth microdilution	0.25–0.5 mg/mL	*S. aureus*	Hermosilla et al. (2026)
Methanolic	Leaves	Overlay bioautography with agar	​	*B. subtilis* *P. aeruginosa* *S. aureus* *E. coli*	Mølgaard et al. (2011)
	Stems	Overlay bioautography with agar	​	*B. subtilis* *P. aeruginosa* *S. aureus* *E. coli*	Mølgaard et al. (2011)
Ethanolic	Leaves	Agar diffusion	ND	*S. aureus* *E. coli*	Pardo et al. (1993)
Hydroalcoholic	Leaves	Broth microdilution	1,024 μg/mL (as TPC expressed as catechin)	*P. aeruginosa*	Araya et al. (2022)
			0.25–0.5 mg/mL	*S. aureus*	Hermosilla et al. (2026)
Essential oil	Leaves	Broth microdilution	100–1,000 μg/mL (MIC: 25 μg/mL)	*P. larvae*	Pellegrini et al. (2017)

ND, not described; IC50, half-maximal inhibitory concentration; TPC, total phenol content; MIC, minimum inhibitory concentration.

Notably, one clinical study has evaluated a *B. globosa* extract in women undergoing nitrofurantoin treatment for urinary tract infections (Letelier et al., 2017). In this trial, patients received the antibiotic along with placebo capsules or capsules of spray-dried standardized hydroalcoholic extract of *B. globosa* leaves (BG126®). Patients treated with BG126® showed negative urine cultures at the end of treatment, whereas positive cultures remained in a proportion of the placebo group. No significant differences in hematological or clinical chemistry parameters were observed between groups, supporting the tolerability of the formulation. This study is particularly relevant because, in contrast to the predominantly *in vitro* evidence available for *B. globosa*, it provides clinical evidence supporting its potential therapeutic application in urinary tract infections. Additionally, BG126® has been formulated as powder (Araya et al., 2022) or included in wound healing polymeric scaffolds (Ceriani et al., 2025; Bahamondez-Canas et al., 2026) showing inhibitory activity against *P. aeruginosa* and *S. aureus*. These studies provide clinical evidence supporting the potential therapeutic application of this plant in urinary tract infections and wound-associated pathogens. These findings further support the translational potential of *B. globosa* in antibacterial applications.

### 3.2 Antibacterial-related activity of compounds identified in *Buddleja globosa*

Based on the available phytochemical profiling of *B. globosa* leaves, this section prioritizes compounds that are consistently reported in the species and that have documented antibacterial activity. These include phenylethanoid glycosides and flavonoid glycosides, particularly verbascoside, isoverbascoside, forsythoside B, luteolin-7-O-glucoside, apigenin-7-O-glucoside, quercetin, and caffeoyl derivatives (Quintero-Pertuz et al., 2025; Houghton, 2003). Although several of these metabolites have demonstrated antibacterial or antibiotic-adjuvant activities, in many cases the available evidence comes from commercially available standards or compounds isolated from other plant species, rather than from compounds purified directly from *B. globosa*. Table 2 summarizes the available evidence for these compounds and provides a qualitative classification based on the studies discussed below. The categories should not be interpreted as a strict cross-study ranking, as MIC values and other activity measures varied according to bacterial species, strain, formulation, and experimental conditions.

**TABLE 2:** Comparative summary of antibacterial and antibacterial-related evidence for principal compounds associated with *Buddleja globosa.*

Compound	Antibacterial potency (MIC range)^a^	Antibiofilm activity	Antivirulence/antitoxin activity	Cytotoxicity	*In vivo* evidence	Overall development priority
Verbascoside	Variable: *S. aureus* (10 to above 512 μg/mL in resistant strains) and *S. epidermidis* (10 μg/mL)	High	High	Moderate (>50 μg/mL in some cells)	Yes	High
Isoverbascoside	Low to moderate: *S. epidermidis* (10 μg/mL) and *S*. *aureus* (625 μg/mL)	NR	NR	NR	NR	Low
Forsythoside B	Moderate: *P. aeruginosa* (32 μg/mL) and *A. baumanii* (64 μg/mL)	NR	High	Favorable	Yes	High
Caffeic acid	Low: 250->1,000 ug/mL	NR	Moderate	NR	Yes	Low
Luteolin	Moderate: *S. aureus* (62.5–125 μg/mL)	Moderate to high	Moderate	NR	Yes	High
Luteolin-7-O-glucoside	Selective; high against some Gram-positive bacteria: *S. aureus* and *E. faecalis* (3 μg/mL); *K. pneumoniae* (25 μg/mL)	NR	NR	NR	No	High but selective
Apigenin	Variable, moderate: *E. coli* and *S. aureus* (20 mg/mL)	Low to moderate	High	Not clearly	Yes	High
Apigenina-7-O-glucoside	Selective; high against some species: *S. aureus* (3 μg/mL), *E. faecalis* (6 μg/mL), *K. pneumoniae* (25 μg/mL)	High	Moderate	Not reported	No	High but selective
Quercetin	Variable, moderate: *S. aureus* (7.75–30 μg/mL), *P. aeruginosa, E. coli and S. entérica* Thyphimurium (20–25 μg/mL)	NR	High	NR	Yes	High
Catechin	Variable, low: *S. aureus* and MRSA (78.1–1,024 μg/mL)	Moderate to high	Limited*	Favorable*	Yes*	High
Spathulenol	Moderate	NR	NR	NR	No	Low
Linalool	Variable, low: 100–3,200 μg/mL	NR	Moderate	NR	Yes*	Moderate
Thymol	Moderate: *S. aureus* (72 μg/mL)	Moderate	Moderate	NR	NR	Moderate
Carvacrol	Moderate: *S. aureus* and *E. coli* (200 μg/mL)	Low	Moderate	NR	Yes	Moderate
α-Terpineol	Low to moderate: 100–16000 μg/mL	NR	NR	NR	NR	Low

MIC, minimum inhibitory concentration; NR, not reported.

^a^ Potency was qualitatively categorized using EUCAST, reference MIC, target ranges as contextual benchmarks: low (>128 μg/mL), moderate (>10–128 μg/mL), and high (≤10 μg/mL).

* When tested as active ingredient of a formulation (in nanospheres, liposomes or micelles carriers).

#### 3.2.1 Verbascoside and isoverbascoside

**FIGURE 3:**
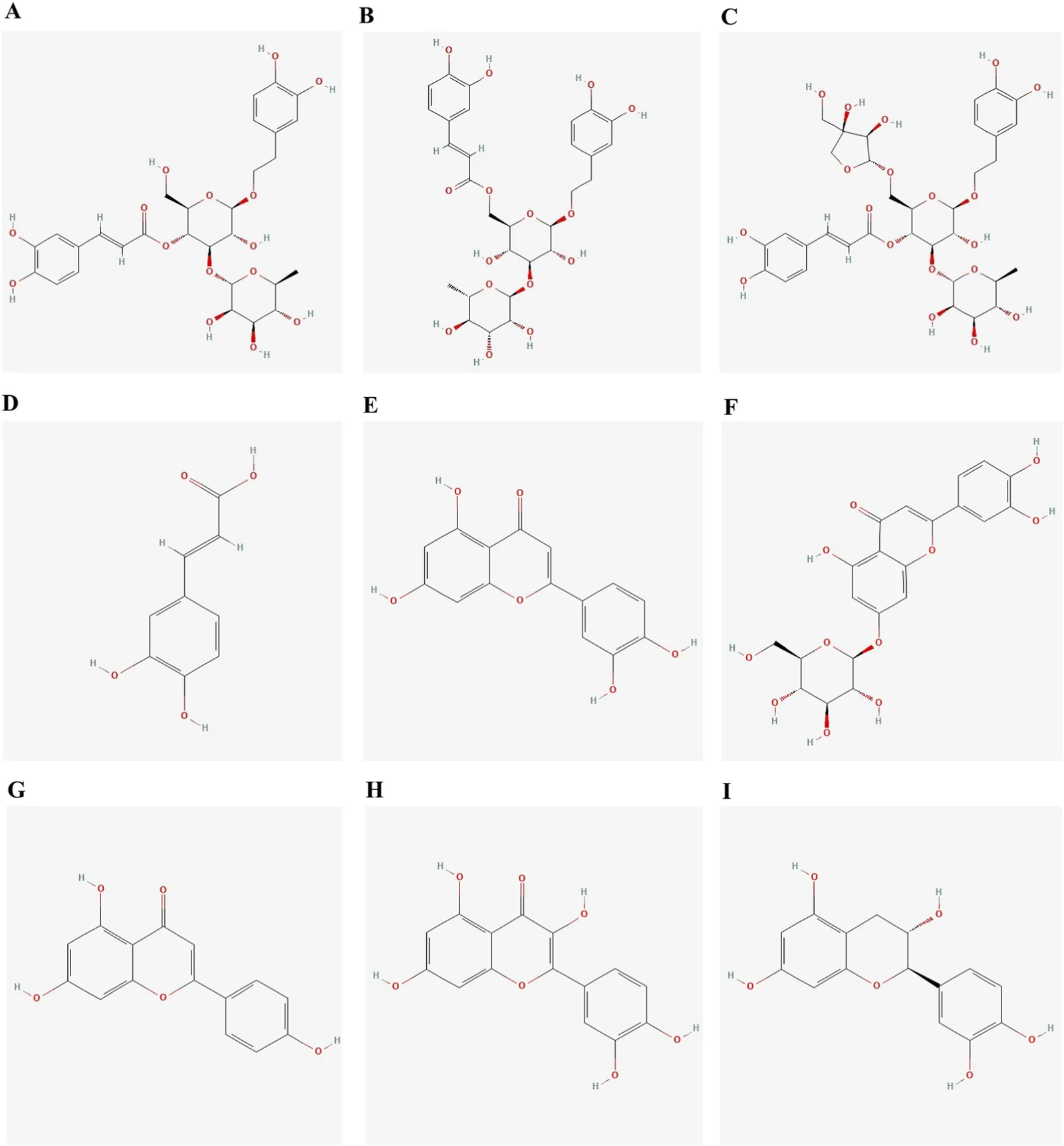
Chemical structures of main compounds isolated from *Buddleja globosa* extracts. **(A)** verbascoside, **(B)** isoverbascoside, **(C)** forsythoside B **(D)** caffeic acid, **(E)** luteolin, **(F)** luteolin 7-O-glucoside, **(G)** apigenin, **(H)** quercetin, and **(I)** catechin. Chemical structure images were obtained from PubChem.

Verbascoside, also known as acteoside (Figure 3A), is consistently reported as one of the major compounds in *B. globosa* and is therefore among the most relevant metabolites for discussing the biological activity of this species (Pardo et al., 1993). Many of the wound healing properties attributed to *B. globosa* extracts have been associated with verbascoside, particularly because of its antioxidant and anti-inflammatory activity (Pongkitwitoon et al., 2024). This compound has been isolated from aqueous and hydroalcoholic leaf extracts, and recent extraction approaches using deep eutectic solvents (DES) have increased its recovery from *B. globosa* leaves (Torres-Vega et al., 2021).

The direct antibacterial activity of verbascoside appears to be variable and strongly dependent on the bacterial species, strain, and experimental conditions. Avila et al. reported that the antibacterial effect of verbascoside on *S. aureus* was associated with inhibition of protein synthesis (Avila et al., 1999). However, reported MIC values against *S. aureus* vary widely, ranging from 10 μg/mL (Agampodi et al., 2022) to values above 200 μg/mL (Fazly et al., 2018), and above 512 μg/mL in antibiotic-resistant strains (Li et al., 2024; Shi et al., 2022). Against *Staphylococcus epidermidis,* an MIC of 10 μg/mL has been reported (Agampodi et al., 2022), whereas MIC values above 256 μg/mL have been described against *Helicobacter pylori, Enterococcus faecalis, E. coli, Proteus vulgaris, A. baumannii,* and *P. aeruginosa* (Agampodi et al., 2022; Kahraman et al., 2018; Rao et al., 2012).

In addition to direct growth inhibition, verbascoside has shown relevant antibiofilm and antivirulence activity. Verbascoside inhibited *S. aureus* biofilm formation at concentrations ranging from 0.625 to 8 μg/mL (Shi et al., 2022). Li et al. proposed that this inhibition of bacterial attachment is related to the ability of verbascoside to inhibit sortase A, an enzyme involved in anchoring surface proteins required for adhesion and colonization. In the same study, verbascoside reduced *S. aureus* infection of alveolar cells *in vitro* and increased the survival of *S. aureus*-infected *Galleria mellonella* larvae (Li et al., 2024). Yang et al. also reported that verbascoside did not reduced *S. aureus* viability but increased the survival of alveolar cells cocultured with *S. aureus* at 4 μg/mL. This protective effect was associated with significant inhibition of the eukaryotic-like serine/threonine phosphatase Stp1, a regulator of *S. aureus* virulence factors (Yang Y. et al., 2021). These findings support the classification of verbascoside as an antivirulence compound in some infection models.

Verbascoside has also demonstrated antitoxin and host-protective effects against other bacterial pathogens. It protected alveolar cells from pneumolysin, a major virulence factor of *S. pneumoniae*, and prevented lung damage while increasing survival in a murine model of pneumococcal pneumonia (Zhao et al., 2016). Similarly, although verbascoside did not show direct antimicrobial activity against *Clostridium perfringens*, it reduced bacterial load and mortality in infected mice and protected colorectal adenocarcinoma cells from the virulence factors α-toxin and perfringolysin O (Zhang et al., 2020). These studies indicate that the anti-infective relevance of verbascoside may depend more on virulence attenuation, toxin neutralization, and host-cell protection than on direct bacterial killing.

Regarding cellular safety, verbascoside did not induce phototoxicity in lung fibroblasts. However, it decreased the viability of lung fibroblasts, keratinocytes, and alveolar cells at concentrations equal to or higher than 50 μg/mL, while no effect was reported in hepatic cells (Shi et al., 2022; Henn et al., 2019).

Isoverbascoside, also known as isoacteoside (Figure 3B), has been less extensively studied than verbascoside but has shown antibacterial activity against selected pathogens. It inhibited *H. pylori* (Rao et al., 2012), and showed activity against *S. epidermidis*, with a reported MIC of 10 μg/mL, whereas its activity against *S. aureus* was lower, with a reported MIC of 625 μg/mL (Agampodi et al., 2022). These findings suggest that isoverbascoside may contribute to the antimicrobial profile of *B. globosa*, although the available evidence remains more limited than for verbascoside.

#### 3.2.2 Forsythoside B

Forsythoside B is a phenylethanoid glycoside identified in *B. globosa* extracts with reported antibacterial-related activity (Figure 3C). Its activity has been associated with bacterial membrane disruption and inhibition of virulence-associated mechanisms. Shi et al. reported MIC values of 32 μg/mL and 64 μg/mL against *P. aeruginosa* and *A. baumannii*, respectively (Shi et al., 2024). In the same study, forsythoside B enhanced the activity of levofloxacin, meropenem, and ceftazidime against multidrug-resistant *P. aeruginosa* and *A. baumannii*, increasing antibiotic efficacy by up to 64-fold. In contrast, lower activity has been reported against *S. aureus*, with MIC values ranging from 128 to 256 μg/mL (Nazemiyeh et al., 2008), indicating that its antibacterial effect may be stronger against selected Gram-negative pathogens than against Gram-positive bacteria.

The biological safety and *in vivo* anti-infective potential of forsythoside B have also been evaluated. Forsythoside B showed low cytotoxicity in keratinocyte, alveolar, and hepatic cell lines, and increased the survival of mice with systemic *P. aeruginosa* infection, although its protective effect was slightly lower than that of ceftazidime (Shi et al., 2024). In addition to direct antibacterial and antibiotic-adjuvant activity, forsythoside B has demonstrated antivirulence or host-protective effects. It protected alveolar cells from pneumolysin, a major pore-forming toxin produced by *S. pneumoniae*, and increased survival in murine models of pneumococcal pneumonia (Wang Z. et al., 2024). Forsythoside B has also improved outcomes in a murine model of sepsis induced by caecal ligation and puncture (Jiang et al., 2012). Together, these findings indicate that forsythoside B displays antibacterial-related activity through multiple mechanisms, including direct bacterial inhibition, antibiotic potentiation, membrane disruption, toxin-associated protection, and host-protective effects during bacterial infection.

#### 3.2.3 Caffeic acid

Caffeic acid (Figure 3D), a phenolic acid detected in *B. globosa* as part of caffeoyl-containing derivatives, has been evaluated for antibacterial-related activity against several bacterial species. Although some studies have reported activity against both Gram-positive and Gram-negative bacteria (Ozçelik et al., 2011), its direct antibacterial potency appears to be variable and, in some cases, relatively low. Xu et al. reported a low inhibitory activity of caffeic acid against *S. aureus, E. coli, Staphylococcus haemolyticus, Staphylococcus chromogenes, K. pneumoniae,* and *P. aeruginosa,* with MIC values from 250 to 1,000 μg/mL (Xu et al., 2022). In *Streptococcus mutans*, caffeic acid showed also weak inhibitory activity, with an MIC of 10 mM (approx. 1802 μg/mL), without detectable toxicity against macrophages at this concentration. Similar MIC values (above 256 μg/mL) were reported for *S. aureus* and *S. epidermidis* by Kępa et al. (Kępa et al., 2025; Kępa et al., 2018). However, caffeic acid showed a significant synergistic effect when combined with selected antibiotics, including norfloxacin (Lima et al., 2016), erythromycin (Kępa et al., 2025), clindamycin, and cefoxitin, indicating potential antibiotic-adjuvant activity against *S. aureus* (Kępa et al., 2018). On *E. coli*, caffeic acid potentiated imipenem activity, and in *P. aeruginosa*, showed synergistic effects with gentamicin and imipenem (Lima et al., 2016).

Beyond direct antibacterial or antibiotic-potentiating effects, caffeic acid has also been reported to enhance host resistance to bacterial infection. In a *Caenorhabditis elegans,* a nematode model commonly used for *in vivo* infection studies, caffeic acid at 3.6 μg/mL improved resistance to *P. aeruginosa* infection and reduced intestinal bacterial burden. The study also reported a significant inhibition of *P. aeruginosa* at the same concentration (Yi et al., 2024). These findings suggest that caffeic acid may contribute to antibacterial-related activity not only through direct bacterial inhibition or antibiotic synergy, but also through host-directed mechanisms that enhance innate immune defenses during bacterial infection.

#### 3.2.4 Luteolin and luteolin-7-O-glucoside

Sato et al. evaluated the antibacterial activity of luteolin (Figure 3E) against methicillin-resistant *S*. *aureus* (MRSA) and methicillin-sensitive *S. aureus* (MSSA), reporting MIC values of 62.5 μg/mL and 125 μg/mL, respectively (Sato et al., 2000). Mechanistic studies suggest that luteolin can disrupt bacterial cell wall and membrane integrity, alter protein expression, and inhibit nucleic acid synthesis in a dose-dependent manner across different bacterial species (Guo et al., 2020). In addition to direct antibacterial activity, luteolin has been reported to suppress biofilm formation by *E. coli*, *Enterobacter cloacae*, *S. aureus*, and *Listeria monocytogenes*, reducing biofilm biomass and supporting its potential role as an antibiofilm compound (Yang et al., 2026; Qian et al., 2020; Qian et al., 2021).

The antimicrobial-related potential of luteolin has also been explored through formulation strategies aimed at overcoming its hydrophobicity and low oral bioavailability. Luteolin-loaded polymeric micelles showed greater activity than free luteolin in bacterial lung infection models (Miao et al., 2021). *In vitro*, these micelles reduced the adhesion of *K. pneumoniae* to lung epithelial cells and enhanced the antibacterial response of macrophages. *In vivo*, the micellar formulation promoted bacterial clearance and reduced inflammatory infiltration in a *K. pneumoniae*-induced pulmonary infection model. These findings suggest that luteolin may exert antibacterial-related effects through a combination of direct antibacterial activity, inhibition of bacterial adhesion, modulation of host antibacterial responses, and formulation-enhanced bioavailability.

Unlike luteolin aglycone, the antibacterial activity of luteolin-7-O-glucoside (also known as cynaroside) appears to be highly species-dependent (Figure 3F). This compound showed strong inhibitory activity against selected Gram-positive bacteria, with reported MIC values of 3 μg/mL against *S. aureus* and *E. faecalis*. It also showed activity against *K. pneumoniae*, with an MIC of 25 μg/mL. In contrast, its activity was markedly lower against *E. coli, P. aeruginosa*, and *Proteus mirabilis*, with MIC values above 1,600 μg/mL (Sezen et al., 2023). Additional evidence from agar diffusion assays also showed moderate inhibitory activity against *K. pneumoniae*, *S*. *enterica* Typhimurium, *P. vulgaris, B. subtilis*, and *E. coli* with inhibition zones under 15 mm (Chiruvella et al., 2007). However, these inhibition-zone results should be interpreted cautiously, since agar diffusion assays are less quantitative than MIC determinations. Overall, these findings suggest that luteolin-7-O-glucoside may contribute to the antibacterial activity of *B. globosa* extracts in a selective manner, with stronger activity against *S. aureus, E. faecalis*, and *K. pneumoniae* than against other Gram-negative pathogens such as *E. coli, P. aeruginosa,* and *P. mirabilis.*

#### 3.2.5 Apigenin and apigenin-7-O-glucoside

Apigenin (Figure 3G) is one of the most extensively studied flavonoids with antimicrobial-related activity, although the reported direct antibacterial potency is variable. Early studies describedbroad antimicrobial activity of against several bacterial species, including *E. coli, P. mirabilis, P. aeruginosa, A. baumannii, K. pneumoniae, E. faecalis, S. aureus,* and *B. subtilis* (Ozçelik et al., 2011). However, some of these effects were observed only at high concentrations. For example, proliferation and adhesion of *E. coli* and *S. aureus* were inhibited by 20 mg/mL of apigenin, which was enhanced when combined with quercetin (Wang Q. et al., 2024). More recent studies reported lower MIC values, including 31.25 μg/mL against Gram-positive bacteria such as *S. aureus* and *B. subtilis,* and 62.5 μg/mL against Gram-negative bacteria such as *E. coli* and *P. aeruginosa* (Liu et al., 2013). In addition, apigenin selectively inhibited DNA gyrase in quinolone-resistant *S. aureus* (Dong et al., 2013), suggesting that its antibacterial activity may depend on the resistance background of the tested strain.

The biological activity of apigenin has also been explored using pharmaceutical formulations. An apigenin hydrogel inhibited *S. aureus* and *E. coli* proliferation and adhesion *in vitro* (Wang Q. et al., 2024), suggesting that formulation strategies may improve the local antimicrobial performance of apigenin, particularly for topical applications. The most consistent evidence for apigenin relates to antivirulence activity. Dong et al. reported that, despite the weak direct antibacterial activity against *S. aureus,* apigenin reduced α-hemolysin production in a dose-dependent manner (Dong et al., 2013). It has also shown to protect against pneumolysin-mediated damage in *S. pneumoniae* infection models (Song et al., 2016), and to inhibit the hemolytic activity of suilysin from *Streptococcus suis* when combined with ampicillin in *in vitro* and *in vivo* models (Lu et al., 2021). Thus, apigenin appears to contribute to infection control mainly through toxin-neutralizing activity and antivirulence mechanisms rather than direct antibacterial killing*,*.

Several mechanisms have been proposed to explain the antibacterial-related effects of apigenin. In *E. coli*, apigenin has been associated with activation of oxidative pathways involving the production and accumulation of reactive oxygen species (ROS) and reactive nitrogen species (RNS), ultimately contributing to bacterial death (Kim et al., 2020). Liu et al. evaluated apigenin and related derivatives, reporting moderate antibacterial activity against Gram-positive bacteria, including *S. aureus* and *B. subtilis*, and Gram-negative bacteria, including *E. coli* and *P. aeruginosa*, with MIC values of 31.25 μg/mL and 62.5 μg/mL, respectively (Liu et al., 2013). Notably, some apigenin derivatives showed stronger antibacterial activity than apigenin itself, suggesting that structural modification may improve the antibacterial potency of this flavonoid.

Among these related compounds, apigenin-7-O-glucoside is particularly relevant because it has been reported in *B. globosa*. In *S. aureus* and *E. coli*, apigenin-7-O-glucoside showed MIC and minimum biofilm inhibitory concentration (MBIC) values below 0.2 mg/mL. Its antibiofilm effect has been associated with quorum-sensing inhibition and reduced EPS production (Lou et al., 2019; Pei et al., 2023). Additional evidence indicates that apigenin-7-O-glucoside has selective antibacterial activity, with reported MIC values of 3 μg/mL against *S. aureus*, 6 μg/mL against *E. faecalis*, and 25 μg/mL against *K. pneumoniae*. In contrast, its activity was markedly lower against *E. coli, P. aeruginosa*, and *P. mirabilis*, with MIC values above 800 μg/mL (Sezen et al., 2023). These findings suggest that apigenin-7-O-glucoside may contribute to the antibacterial-related activity of *B. globosa* extracts in a species-dependent manner, with stronger activity against selected Gram-positive bacteria and *K. pneumoniae* than against other Gram-negative pathogens.

#### 3.2.6 Quercetin

Quercetin (Figure 3H) has been the focus of numerous studies highlighting its antibacterial activity against *S. aureus*. Veiko et al. revealed that quercetin exhibits varying degrees of antibacterial activity depending on the concentration, successfully inhibiting the growth of *S. aureus* at 100 µM (equivalent to 30.2 μg/mL) (Veiko et al., 2023). However, the literature reports variable MIC values, making it difficult to define a consistent antimicrobial threshold for this compound. In MRSA, quercetin has shown lower MIC values, reaching 7.75 μg/mL, which suggests greater activity against some resistant *S. aureus* strains (Güran et al., 2019).Other studies on *S. aureus*, *P. aeruginosa, E. coli, and S. enterica* Typhimurium, report MIC values in a narrower range from 20 to 25 μg/mL (Jaisinghani, 2017; Basile et al., 2000; Wang et al., 2018).

Mechanistically, quercetin has been associated with damage to the cell wall and membrane of *S. aureus* and *E. coli* (Wang et al., 2018). Its antimicrobial activity has also been documented against clinical strains of MRSA, vancomycin-intermediate *S. aureus*, cefoxitin-resistant *Staphylococcus saprophyticus*, vancomycin-resistant *S. aureus,* and oxacillin- and vancomycin-resistant *S. saprophyticus*.

In addition to its direct antibacterial activity, quercetin was shown to inhibit pneumolysin, a major virulence determinant produced by *S. pneumoniae* (Lv et al., 2020). This mechanism is particularly relevant because pneumolysin released after bacterial killing by conventional antibiotics may continue to damage host tissues. In a murine model of endonasal pulmonary infection, quercetin improved survival after lethal *S. pneumoniae* challenge, reduced lung tissue damage, and decreased the release of inflammatory cytokines. These findings suggest that quercetin may contribute to pneumococcal infection control through toxin neutralization and host-protective effects, rather than only through direct bacterial growth inhibition.

#### 3.2.7 Catechin

Sinsiwar and Vadivel (Sinsinwar and Vadivel, 2020) and Gomes et al. (Gomes et al., 2018) investigated catechin (Figure 3I) against *S. aureus* and MRSA strains and reported MIC values ranging from 78.1 to 1,024 μg/mL. The antibacterial effect was associated with disruption of MRSA membrane integrity, increased ROS production, and decreased antioxidant enzyme activity, suggesting that oxidative stress and membrane damage contribute to bacterial inhibition.

When combined with antibiotics, catechin showed modulatory effects that varied according to the bacterial species and antibiotic tested. Catechin significantly reduced the MIC of norfloxacin and gentamicin against *S. aureus*, as well as the MIC of imipenem, tetracycline, and erythromycin against *E. coli*, suggesting a potentiating effect in these combinations (Gomes et al., 2018). However, catechin increased the MIC of tetracycline against *S. aureus* and erythromycin against *P. aeruginosa*. The adjuvant potential of catechin has also been evaluated *in vivo* (Lee et al., 2005). In a rat model of chronic bacterial prostatitis induced by *E. coli*, catechin alone showed a trend toward reduced bacterial growth, although these effects were not statistically significant. In contrast, the combination with ciprofloxacin significantly reduced bacterial growth and improved inflammatory findings compared with the untreated control and with ciprofloxacin alone groups. These results support the potential of catechin as an adjuvant to antibiotic therapy, particularly in infection models where combined treatment may improve antibacterial and anti-inflammatory outcomes.

The therapeutic application of catechin may be limited by its poor aqueous solubility. To overcome these limitations, the same research group developed a catechin-based liposomal system (Sinsinwar and Vadivel, 2021). This formulation improved the antibacterial activity of catechin compared with non-formulated catechin systems, reducing the MIC against MRSA. In a murine surgical site infection model, the formulation reduced bacterial burden and attenuated excessive inflammation in infected skin tissue. More recently, catechin-loaded polymeric nanospheres were evaluated against *S. pneumoniae* and MRSA, showing MIC values of 130 μg/mL and 128 μg/mL, respectively. This nanosystem reduced biofilm formation by 83% and inhibited bacterial growth while maintaining high fibroblast cell viability *in vitro*. Together, these studies suggest that catechin displays moderate and variable direct antibacterial activity, particularly against MRSA, but that its anti-infective potential may be substantially improved through antibiotic combination strategies and formulation approaches that enhance solubility, delivery, antibiofilm performance, and local tissue compatibility.

#### 3.2.8 Volatile terpenoids and other minor antibacterial compounds

Spathulenol has demonstrated moderate to high antibacterial activity against various strains, including clinical isolates of *Mycobacterium tuberculosis* (Do Nascimento et al., 2018; Dzul-Beh et al., 2019). Linalool has been tested against various bacterial species, such as *E. coli* and *S. aureus*, but the reported activity was low (MIC ranging from 100 to 3,200 μg/mL) (Park et al., 2012; Guimarães et al., 2019). Mechanistically, linalool has been associated with oxidative stress, lipid peroxidation, protein degradation, membrane damage, leakage of nucleic acids, and inhibition of respiratory chain activity through decreased dehydrogenase activity (Yang SK. et al., 2021; Guo et al., 2021; Liu et al., 2020). Linalool-loaded microcapsules also reduced *P. aeruginosa* virulence and microbial load *in vitro* and in a murine wound infection model (Kanekar et al., 2022), suggesting that formulation strategies may enhance its antibacterial-related performance.

Thymol has shown antibacterial activity against *S. aureus* and *E. coli* with variable MIC values from 7 to 72 μg/mL (Guimarães et al., 2019), and antibiofilm activity at concentrations between 20 and 200 μg/mL, with higher concentrations required to disrupt mature biofilms (Sousa et al., 2020). It has also been described as an antivirulence compound through inhibition of the Type III secretion system-1 in *S. enterica* (Shu et al., 2016). Carvacrol has also shown activity against *S. aureus* and *E. coli* with MIC values from 15 to 200 μg/mL, although higher concentrations were required to eradicate established *S. aureus* biofilms (Guimarães et al., 2019; García-Salinas et al., 2018). It has also shown activity against *P. aeruginosa*, clinical isolates of *S. aureus*, and carbapenem-resistant and polymyxin-resistant *K. pneumoniae*. In addition, carvacrol reduced biofilm biomass and metabolic activity in *S. enterica* Typhimurium, inhibited *Campylobacter jejuni* motility at subinhibitory concentrations, reduced intestinal pathogen loads in a murine model of campylobacteriosis, and increased MRSA susceptibility to β-lactam antibiotics (Trevisan et al., 2018; van Alphen et al., 2012; Mousavi et al., 2020). Therefore, carvacrol is better interpreted as a multifunctional antibacterial-related monoterpene, with direct antibacterial, antibiofilm, antivirulence, and antibiotic-adjuvant effects.

Other monoterpenes, such as α-terpineol, have also been evaluated against bacterial species, including cariogenic and periodontopathic bacteria, with reported MIC values between 100 and 1,600 μg/mL (Park et al., 2012). Wijayati et al. (Wijayati et al., 2016), produced α-terpineol, showing that it exhibited better inhibition against *Bacillus megaterium* compared to *P. aeruginosa*. Jirovetz et al. demonstrated that this monoterpene had antimicrobial activity against both Gram-positive and Gram-negative bacteria (Jirovetz et al., 2005).

### 3.3 Antibiotic potentiation by compounds identified in *Buddleja globosa*

The identification and applicability of synergism in antimicrobial agents can significantly enhance the antimicrobial efficacy of current antibiotics. Synergistic activity is usually expressed as reduction in the MIC of the respective antibiotic or by determining the fractional inhibitory concentration (FIC) or FIC index (FICI). While the decrease in MIC only reflects the effect on this antibiotic parameter, the FICI also considers the impact of the combination on the second compound. Table 3 summarizes the reports of synergism with these compounds organized by pathogens.

**TABLE 3:** *Buddleja globosa* compounds with reported synergism with antibiotics.

Species	Antibiotic	Compound	MIC decrease or FICI*	Ref.
*A. baumannii*	ciprofloxacin	catechin	3x	Ibitoye and Ajiboye (2019)
	gemifloxacin	catechin	4x	
MDRAB	ceftazidime levofloxacin meropenem	forsythoside B forsythoside B forsythoside B	2 to 16x 2 to 32x 8 to 16x	Shi et al. (2024)
*P. aeruginosa*	erythromycin	apigenin	0.375*	Zhang et al. (2019)
MDRPA	ceftazidime ceftazidime gentamycin imipenem levofloxacin meropenem	forsythoside B verbascoside caffeic acid caffeic acid forsythoside B forsythoside B	16 to 64x 0.281 25x 16x 4 to 16x 2 to 8x	Shi et al. (2024) Shi et al. (2022) Lima et al. (2016) (Shi et al., 2024)
*S. aureus*	cefazolin	terpinen-4-ol	0.5*	Cordeiro et al. (2020)
	cefoxitin	caffeic acid	2x	Kępa et al. (2018)
	cefoxitin	catechin	2x to 128x	Miklasińska et al. (2016)
	clindamycin	caffeic acid	2x	Kępa et al. (2018)
	clindamycin	catechin	2x	Miklasińska et al. (2016)
	erythromycin	catechin	2x to 64x	Kępa et al. (2018)
	gentamicin	caffeine	2x	Fazly et al. (2018)
	gentamicin	catechin	2x	Gomes et al. (2018)
	gentamicin	verbascoside	2x	Fazly et al. (2018)
	meropenem	terpinen-4-ol	0.5*	Cordeiro et al. (2020)
	norfloxacin	catechin	17x	Gomes et al. (2018)
	oxacillin	terpinen-4-ol	0.32*	Cordeiro et al. (2020)
	sulfamethoxazole vancomycin	quercetin verbascoside	2x 0.375*	Sahyon et al. (2019) Shi et al. (2022)
MRSA	ampicillin ampicillin	apigenin carvacrol	7.5x <0.5*	Akilandeswari and Ruckmani (2017) Li et al. (2023)
	cefotaxime	lupeol	4x	Okusa et al. (2014)
	cefotaxime ceftriaxone	carvacrol apigenin	<0.5* 22x	Li et al. (2023) Akilandeswari and Ruckmani (2017)
	gentamicin norfloxacin	verbascoside caffeic acid	2x 10x	Fazly et al. (2018) Lima et al. (2016)
	oxacillin	carvacrol	<0.5*	Li et al. (2023)
	penicillin	lupeol	8x	Okusa et al. (2014)
	streptomycin tetracycline doxycycline chlortetracycline vancomycin	lupeol linalool linalool linalool verbascoside	4x 2x 4x 8x 0.095*	Okusa et al. (2014) Wen et al. (2026) Wen et al. (2026) Wen et al. (2026) Shi et al. (2022)
*S*. *enterica* Typhimurium	sulfamethoxazole	quercetin	4x	Sahyon et al. (2019)
*K. pneumoniae* carbapenemase- producing	meropenem	linalool	0.5*	Yang et al. (2021b)
*E. coli*	erythromycin	catechin	2x	Gomes et al. (2018)
	gentamicin	verbascoside	2x	Fazly et al. (2018)
	imipenem	catechin	4x	Gomes et al. (2018)
	colistin	apigenin	4 to 32x 0.3–0.6*	Feng et al. (2024)
	sulfamethoxazole	quercetin	2x	Sahyon et al. (2019)
MDREC	imipenem	caffeic acid	1.5x	Lima et al. (2016)

* FICI, fractional inhibitory concentration index; MDRAB, Multidrug resistant *A. baumanii*; MDRPA, Multidrug resistant *P. aeruginosa*; MRSA, Methicillin-resistant *S. aureus*; MDREC, Multidrug resistant *E. coli*.

Among the compounds identified in *B. globosa*, forsythoside B shows one of the most relevant antibiotic-potentiating profiles. In multidrug-resistant *P. aeruginosa* and *A. baumannii*, forsythoside B enhanced the activity of clinically used antibiotics, including ceftazidime, levofloxacin, and meropenem, with reported MIC reductions of up to 64-fold (Shi et al., 2024). This evidence is particularly important because forsythoside B is a phenylethanoid glycoside reported in *B. globosa* extracts and has also shown direct antibacterial activity and low cytotoxicity in relevant cell lines. Therefore, forsythoside B should be highlighted as a promising antibacterial-adjuvant candidate among the metabolites most closely linked to the phytochemical profile of *B. globosa*.

Verbascoside has also shown antibiotic-potentiating activity, although the reported effects appear more moderate than those of forsythoside B. Verbascoside reduced the effective concentration of selected antibiotics against *P. aeruginosa, S. aureus,* MRSA, and *E. coli,* including combinations with ceftazidime, gentamicin, and vancomycin. Because verbascoside is abundant in *B. globosa* and has additional antibiofilm, antivirulence, and antitoxin effects, its antibiotic-potentiating activity should be interpreted as part of a broader antibacterial-related profile rather than as an isolated synergistic effect.

Caffeic acid also deserves attention because its direct antibacterial activity is generally weak or variable, with several reported MIC values above 256 μg/mL, but its antibiotic-potentiating effects are more consistent. Caffeic acid reduced the MIC of selected antibiotics against *S. aureus*, including norfloxacin, erythromycin, clindamycin, and cefoxitin. It also potentiated imipenem activity against *E. coli* and showed potentiating effects with gentamicin and imipenem against *P. aeruginosa* (Kępa et al., 2025; Kępa et al., 2018; Lima et al., 2016). These findings suggest that caffeic acid may be more relevant as an antibiotic adjuvant than as a direct antibacterial compound.

Catechin has one of the broadest sets of antibiotic-modulating evidence, although its interaction with antibiotics is species- and drug-dependent. Catechin reduced the MIC of norfloxacin and gentamicin against *S. aureus*, and of imipenem, tetracycline, and erythromycin against *E. coli* (Gomes et al., 2018). Its adjuvant potential is nevertheless supported by *in vivo* evidence, since catechin combined with ciprofloxacin significantly reduced bacterial growth and improved inflammatory findings compared with ciprofloxacin alone in a rat model of chronic bacterial prostatitis induced by *E. coli* (Lee et al., 2005).

Flavonoids such as apigenin and quercetin also contribute relevant antibiotic-potentiating evidence. Apigenin reduced the MIC of ceftriaxone against MRSA and has also been described as a colistin adjuvant by interacting with MCR-1, a resistance protein involved in colistin resistance, thereby restoring colistin susceptibility in *E. coli*. Quercetin has shown potentiating effects with sulfamethoxazole against *S. enterica* Thyphimurium and *E. coli*. These findings support the potential of flavonoids as antibiotic-adjuvant compounds, although many studies used commercial standards or compounds isolated from sources other than *B. globosa*. Finally, selected terpenoids and other minor compounds have also shown antibiotic-potentiating effects. Carvacrol increased MRSA susceptibility to β-lactam antibiotics, including ampicillin, cefotaxime, and oxacillin. Linalool showed potentiating effects with tetracycline, doxycycline, and chlortetracycline against MRSA, and with meropenem against carbapenemase-producing *K. pneumoniae.* Terpinen-4-ol and lupeol have also shown synergy or MIC reduction with selected antibiotics. However, these compounds should be considered supporting evidence unless their presence and relative abundance in *B. globosa* extracts or essential oil are clearly demonstrated.

Overall, the antibiotic-potentiating evidence reinforces the interpretation that several *B. globosa*-associated metabolites may be more useful as antibacterial adjuvants than as classical direct antibacterial agents. Forsythoside B, verbascoside, caffeic acid, catechin, apigenin, and selected terpenoids provide evidence of improved antibiotic activity in specific combinations.

## 4 Discussion

### 4.1 Strengths and limitations of the available evidence

The evidence reviewed indicates that *B. globosa* extracts and several compounds identified in this species have antibacterial-related potential. However, the strength of this evidence depends on how antibacterial activity is defined. Direct inhibitory or bactericidal activity is usually interpreted through MIC and MBC values, but these parameters should not be evaluated in isolation. Many reported MIC values for natural compounds are above 256 μg/mL, which can be experimentally useful for screening and mechanistic studies, but may have limited pharmacological relevance unless they are compared with the MIC values of current antibiotics, achievable local concentrations, and the cytotoxicity profile of the compound.

To place these MIC values in pharmacological context, it is useful to compare them with standardized reference ranges reported for conventional antibiotics. The EUCAST discussion document on MIC determination includes target MIC values for more than 60 antibiotics tested against laboratory strains of *E. coli, P. aeruginosa, S. aureus*, and *E. faecalis* (European Committee for Antimicrobial Susceptibility Testing EUCAST of the European Society of Clinical Microbiology and Infectious Diseases ESCMID, 2003). Across that dataset, MIC values extend from approximately 0.004 μg/mL to 128 μg/mL, with many values falling below 1 μg/mL for antibiotics such as ciprofloxacin, penicillin, meropenem, or tobramycin. This contrast highlights that, although some compounds associated with *B. globosa* exhibit measurable direct antibacterial activity, their MIC values are often higher and more variable than those typically reported for conventional antibiotics under standardized conditions. Therefore, the pharmacological relevance of these natural compounds should not be judged only by the presence of antibacterial activity, but also by whether the reported MIC values are likely to be achievable at the target site, by the limitations imposed by bioavailability and formulation, and by the available safety margin. For this reason, compounds with modest or variable direct antibacterial potency may still be pharmacologically relevant when they display antibiofilm, antivirulence, host-protective, or antibiotic-potentiating effects, particularly in topical or local delivery settings where higher local concentrations may be achievable.

Most studies reviewed used isolated or commercial compounds and broth dilution methods, particularly microdilution, which provides quantitative MIC data and allows comparison across bacterial species. Nevertheless, relatively few studies complemented antibacterial testing with cytotoxicity assays in relevant human cells, such as keratinocytes, fibroblasts, epithelial cells, hepatic cells, or immune cells. This is a critical limitation because the biological relevance of an antibacterial concentration depends on whether it falls within a safe concentration range. Without this comparison, a compound may appear active *in vitro* but lack a practical therapeutic window. In addition, the quality of the available evidence is heterogeneous. Some compounds are supported by multiple complementary observations, including MIC determinations, antibiofilm or antivirulence assays, cytotoxicity assessment, and *in vivo* validation. In contrast, other findings remain preliminary because they are based on single *in vitro* studies, limited bacterial panels, and high effective concentrations, or the absence of safety and pharmacokinetic context. Therefore, the evidence summarized in this review should be interpreted with different levels of confidence depending on the experimental depth and translational relevance of each report.

Overall, the evidence supporting direct antibacterial activity of many compounds associated with *B. globosa* remains inconsistent, particularly when antibacterial activity is defined strictly as direct inhibition of bacterial growth or viability. In contrast, stronger and more consistent evidence supports antibacterial-related activity, understood here as indirect mechanisms that may contribute to infection control without necessarily cause direct bacterial killing. These include antibiofilm effects, antivirulence actions, host-protective responses, and antibiotic-adjuvant properties. Therefore, the antibacterial relevance of *B. globosa* should not be interpreted solely in terms of direct growth inhibition, but also in light of its potential to interfere with bacterial pathogenicity and improve therapeutic outcomes through complementary infection-modulating mechanisms.

### 4.2 Verbascoside as the most representative antibacterial-related metabolite of *Buddleja globosa*

Among the compounds identified in *B. globosa*, verbascoside is the most representative metabolite for discussing the antibacterial-related potential of this species. This is not because it shows the strongest direct antibacterial potency, but because it is one of the most abundant and consistently reported marker compounds in *B. globosa* extracts and has been evaluated across several antibacterial-related mechanisms. Therefore, verbascoside provides the best connection between the phytochemical identity of *B. globosa* and its potential anti-infective properties.

The direct antibacterial activity of verbascoside should be interpreted cautiously. Although some studies report low MIC values against selected Gram-positive bacteria, including *S. aureus* and *S. epidermidis*, other studies report MIC values above 200, 256, or 512 μg/mL, particularly against antibiotic-resistant strains and several Gram-negative species. These values suggest that verbascoside should not be presented as a uniformly potent direct antibacterial compound. Accordingly, the evidence for verbascoside is stronger in the context of antibiofilm, antivirulence, and host-protective mechanisms than as a direct antibacterial agent. This conclusion is supported by the convergence of multiple mechanistic studies, whereas the direct MIC evidence remains more heterogeneous.

The strongest antibacterial-related evidence for verbascoside is associated with mechanisms beyond direct bacterial killing. Verbascoside has been reported to inhibit *S. aureus* biofilm formation at low concentrations and to interfere with bacterial adhesion. It has also been shown to protect alveolar cells infected with *S. aureus* without reducing bacterial viability, an effect associated with inhibition of Stp1, a regulator of bacterial virulence factors. These findings support the interpretation of verbascoside as an antivirulence compound in selected infection models. Verbascoside has also shown antitoxin and host-protective effects. This suggests that verbascoside may contribute to infection control by reducing bacterial pathogenicity and protecting host tissues rather than by acting only as a conventional antibacterial agent. However, its safety window should be considered carefully, since reductions in the viability of lung fibroblasts, keratinocytes, and alveolar cells have been reported at concentrations equal to or above 50 μg/mL.

Taken together, verbascoside should be highlighted as the most representative antibacterial-related metabolite of *B. globosa*. Its relevance lies in the convergence of abundance in *B. globosa*, antibiofilm activity, antivirulence mechanisms, toxin-associated protection, and host-cell protection. In contrast, forsythoside B may be considered a particularly promising complementary metabolite because it shows stronger direct activity against selected Gram-negative pathogens, potentiates antibiotics against multidrug-resistant *P. aeruginosa* and *A. baumannii*, shows low cytotoxicity in relevant cell lines, and improves outcomes in bacterial infection models. Thus, the strongest translational message is that phenylethanoid glycosides from *B. globosa*, especially verbascoside and forsythoside B, are more promising as multifunctional antibacterial-related agents than as classical broad-spectrum antibiotics.

### 4.3 Direct antibacterial activity versus antibacterial-related mechanisms

The antibacterial relevance of compounds identified in *B. globosa* should be interpreted by distinguishing direct antibacterial activity from broader antibacterial-related mechanisms. Direct activity is usually supported by MIC or MBC values, which indicate inhibition or killing of planktonic bacteria. However, several compounds reviewed here showed MIC values above 256 μg/mL against clinically relevant bacteria. Although these values can be useful for initial screening and mechanistic laboratory studies, they should not be interpreted as strong antibacterial potency by themselves. Their relevance should be balanced against the MIC values of conventional antibiotics, the concentration that could realistically be achieved at the infection site, and the cytotoxicity profile of the compound in relevant mammalian cells.

Only a limited number of compounds showed direct inhibitory activity at concentrations below 10 μg/mL. Among the strongest examples, luteolin-7-O-glucoside showed MIC values of 3 μg/mL against *S. aureus* and *E. faecalis*, while quercetin showed an MIC as low as 7.75 μg/mL against MRSA. Apigenin-7-O-glucoside also showed selective direct antibacterial activity, with reported MIC values of 3 μg/mL against *S. aureus* and 6 μg/mL against *E. faecalis*. These low MIC values support the potential direct antibacterial relevance of these flavonoid glycosides and quercetin against selected Gram-positive bacteria. However, this activity was not broad-spectrum, since the same glycosides showed much weaker activity against several Gram-negative bacteria, including *E. coli, P. aeruginosa*, and *P. mirabilis*. Therefore, the most potent direct inhibitory effects appear to be species-dependent and should be interpreted in relation to the tested bacterial strain and assay conditions.

This distinction is particularly important for phenolic compounds and flavonoids, whose reported direct antibacterial activity is often variable. Verbascoside, caffeic acid, catechin, quercetin, luteolin, and apigenin have all shown some degree of direct inhibitory activity, but their potency varies considerably depending on the bacterial species, strain, and assay conditions. Therefore, these compounds should not be presented as uniformly potent antibacterial agents. In general, the strongest evidence in this review corresponds to compounds or mechanisms supported by low or selective MIC values, mechanistic confirmation, cytotoxicity assessment, and, ideally, *in vivo* validation. By contrast, evidence should be considered preliminary when it is based only on a single *in vitro* report, high effective concentrations, or the absence of safety and translational context.

In this sense, the strongest evidence for several compounds is not necessarily direct bacterial killing, but the modulation of bacterial pathogenicity. Verbascoside is a representative example. Although its direct antibacterial effect is variable, it inhibited *S. aureus* biofilm formation at concentrations ranging from 0.625 to 8 μg/mL, interfered with bacterial attachment through sortase A inhibition, inhibited Stp1-related virulence regulation, and protected host cells from bacterial toxins. Similarly, quercetin and apigenin have shown antitoxin or antivirulence effects by reducing pneumolysin-mediated injury or decreasing α-hemolysin production. These mechanisms are relevant because they may reduce tissue damage and bacterial pathogenicity even when the compound does not strongly inhibit bacterial growth.

Antibiofilm activity also represents an important antibacterial-related mechanism. Biofilm-associated bacteria are generally less susceptible to antibacterial agents than planktonic cells because the extracellular matrix limits penetration, favors persistence, and protects bacteria from host defenses. Therefore, compounds that inhibit adhesion, reduce biofilm biomass, interfere with extracellular polymeric substance production, or disrupt mature biofilms may have therapeutic relevance even when their planktonic MIC values are moderate. In this review, luteolin, apigenin-7-O-glucoside, catechin formulations, thymol, carvacrol, and other compounds showed evidence of antibiofilm activity, supporting the idea that inhibition of colonization and persistence may be one of the most relevant antibacterial-related contributions of these metabolites. Another relevant mechanism is antibiotic potentiation. Several compounds identified in or associated with *B. globosa* reduced the MIC of conventional antibiotics in selected bacterial species. Forsythoside B enhanced the activity of levofloxacin, meropenem, and ceftazidime against multidrug-resistant *P. aeruginosa* and *A. baumannii*, while caffeic acid, catechin, apigenin, and carvacrol also showed antibiotic-modulating effects in specific combinations. However, these interactions should be interpreted cautiously because potentiation is species- and antibiotic-dependent, and some combinations may be neutral or even antagonistic. For this reason, future studies should report fractional inhibitory concentration indices, time-kill kinetics, or other standardized synergy parameters rather than relying only on MIC reduction.

Overall, the evidence suggests that the antibacterial potential of *B. globosa*-associated metabolites is better understood as antibacterial-related activity rather than as classical broad spectrum antibiotic activity. Direct inhibition is relevant when supported by low MIC values and an adequate safety window, as observed for selected flavonoids and flavonoid glycosides against specific Gram-positive bacteria. However, many of the most promising results involve antibiofilm effects, antivirulence mechanisms, toxin neutralization, host-cell protection, and antibiotic adjuvancy. This interpretation provides a more realistic translational framework for *B. globosa*, especially for topical infections, biofilm-associated infections, and combination strategies with existing antibiotics.

### 4.4 Future directions

The studies reviewed support the antibacterial-related potential of *B. globosa* extracts and several metabolites identified in this species. However, future research on *B. globosa*-based antimicrobial strategies should move beyond descriptive antibacterial screening toward a more integrated translational framework focused on the metabolites most consistently discussed in this review. Standardized phytochemical characterization should prioritize the quantification of key compounds identified or reported in *B. globosa*. These markers should be quantified using validated analytical methods and linked to plant organ, harvest season, extraction method, and processing conditions to reduce batch-to-batch variability and improve comparability between studies.

A second priority is to interpret MIC (and MBC) values in a pharmacologically meaningful way. Many reported inhibitory concentrations for natural compounds are above 256 μg/mL. Although these values can be useful for preliminary screening and mechanistic studies, they should not be considered strong antibacterial activity unless they are compared with current antibiotics, achievable concentrations at the infection site, and cytotoxicity thresholds in relevant mammalian cells. Therefore, future studies should report MIC, MBC, MBIC, and MBEC values together with appropriate antibiotic controls and, when possible, selectivity indices that relate antibacterial activity to mammalian cell safety. Cytotoxicity assessment should become a routine part of the evaluation of extracts and isolated compounds. For example, the studies of Mensah et al. (2001) and Hermosilla et al. (2026) tested the viability of fibroblasts exposed to aqueous and hydroalcoholic extracts of *B. globosa.* Both groups found that low concentrations either increase the cell growth or had no impact on viability (equal or lower than 1 μg/mL) while a marked reduction in viability was observed at higher doses (50–500 μg/mL). Similar results were obtained in Chinese hamster ovary (CHO-K1) cells at concentrations above 10 μg/mL) (Zamorano-Aguilar et al., 2020). With these values in perspective, some of the antimicrobial activities summarized in Table 1 may fall within a potentially will be relevant without cytotoxic effects. Future studies should prioritize cell types that are relevant to the intended use, such as keratinocytes and fibroblasts for wound applications, urothelial cells for urinary tract infections, and airway epithelial cells or macrophages for respiratory infections.

Mechanistic validation should also be expanded to clarify whether the antibacterial-related effects of *B. globosa* extracts and associated metabolites are due to direct growth inhibition, membrane disruption, antibiofilm activity, quorum-sensing interference, virulence-factor modulation, host-cell protection, or antibiotic potentiation. In this context, omics-based approaches, including transcriptomics, proteomics, metabolomics, and microbiome-oriented analyses, could help identify bacterial pathways and host responses affected by *B. globosa* extracts or their major metabolites. Structure-activity relationship studies are also needed to compare related compounds, such as phenylethanoid glycosides, flavonoid aglycones and glycosides, phenolic acid derivatives, catechin-related compounds, and selected terpenoids, in order to clarify which structural features contribute to antibacterial, antibiofilm, antivirulence, or antibiotic-adjuvant effects. In addition, pharmacokinetic and pharmacodynamic investigations should evaluate stability, metabolism, tissue exposure, local bioavailability, and safety, particularly for topical, wound-healing, urinary, or mucosal applications. Formulation development should also be prioritized to improve solubility, stability, controlled release, local delivery, and compatibility with infected tissues.

Finally, more *in vivo* and clinical studies are needed. The clinical evaluation of BG126® as a complementary therapy with nitrofurantoin in urinary tract infection and the development of *B. globosa*-loaded wound-healing formulations provide relevant translational precedents. However, these studies remain limited compared with the amount of *in vitro* evidence available. Future research should prioritize infection models that match the traditional and potential therapeutic uses of *B. globosa,* particularly infected wounds, urinary tract infections, and biofilm-associated infections.

Therefore, future research should prioritize standardized toxicity assessments, mechanistic studies, pharmacological characterization, and validation in relevant *in vivo* models to better establish the therapeutic potential and translational applicability of *B. globosa*-derived antimicrobial agents.

## 5 Conclusion

The available evidence supports *B. globosa* as a relevant source of antibacterial-related bioactive compounds. Extracts obtained from this species, particularly polar leaf extracts, have shown activity against bacterial pathogens such as *S. aureus, E. coli*, and *P. aeruginosa*, although the reported potency varies according to plant material, extraction method, phytochemical composition, and antimicrobial susceptibility assay. Among the compounds identified in *B. globosa*, verbascoside and forsythoside B appear to be the most relevant phenylethanoid glycosides. Verbascoside is especially important because of its abundance in the plant and its reported antibiofilm, antivirulence, antitoxin, and host-protective effects, even though its direct antibacterial activity is variable. Forsythoside B is also particularly promising because it combines direct activity against selected Gram-negative pathogens, antibiotic-potentiating effects, low cytotoxicity in relevant cell lines, and *in vivo* anti-infective evidence.

Future studies should prioritize standardized phytochemical characterization of *B. globosa* extracts, quantitative antibacterial testing, comparison with clinically used antibiotics, cytotoxicity assessment in relevant mammalian cells, and validation in *vivo* infection models. These approaches will be essential to determine whether *B. globosa* extracts or their main metabolites can be developed as antibacterial adjuvants, antibiofilm agents, antivirulence compounds, or topical anti-infective formulations.
