# Supplementary material for: Antibacterial properties of Buddleja globosa: a review of extracts and bioactive compounds
Source: Front Pharmacol. 2026 Aug 18;17:1896014. doi: 10.3389/fphar.2026.1896014 (PMC13531183; doi:10.3389/fphar.2026.1896014)
Supplement: Supplementary file 1 [file Table1.docx]

Supplementary Material

**Supplementary Table 1.** Reported phytochemical constituents of B. globosa identified in the literature and categorized by phytochemical group. Compounds highlighted in bold have reported antimicrobial activity and are further discussed in the main manuscript.

| **Classification** | | **Compounds** | **References** |
| --- | --- | --- | --- |
| Polyphenols | Flavonoids | 6-hydroxyluteolin-7-O-glucoside, **apigenin-7-O-glucoside**, **luteolin-7-O-glucoside**, quercetin-3-O-glucoside | (Torres-Vega et al., 2021) |
|  |  | **catechin**, epicatechin | (Zamorano-Aguilar et al., 2020) |
|  |  | scutellarein-7-O-glucoside, rutin | (Houghton, 2013) |
|  |  | 6-hydroxyluteolin, acacetin 7-O-rutinoside, **luteolin** | (Bustamante et al., 2015; Houghton, 2013) |
|  |  | **apigenin**, scutellarein, **quercetin** | (Bustamante et al., 2015) |
|  | Non flavonoids | **caffeic acid**, caffeoylshikimic acid | (Torres-Vega et al., 2021) |
| Phenylethanoid and phenylpropanoids | | **verbascoside** (acteoside) | (Bustamante et al., 2015; Houghton, 2003; Torres-Vega et al., 2021) |
|  |  | β-hydroxyverbascoside, β-hydroxyisoverbascoside, campneoside I, eukovoside, **forsythoside B**, forsythoside A, **isoverbascoside**, lipedoside A, martynoside | (Torres-Vega et al., 2021) |
|  |  | echinacoside | (Houghton, 2003) |
| Sterols | | chondrilasterol | (Bustamante et al., 2015) |
|  |  | β-sitosterol, campesterol, ergosterol, stigmastanol, stigmasterol | (Backhouse et al., 2008) |
| Terpenoids | Monoterpenes | aucubin, catalpol | (Bustamante et al., 2015; Houghton, 2003) |
|  |  | *p-*methoxycinnamoyl aucubin, *p*-methoxycinnamoyl catalpol, catalposide, methylcatalpol | (Houghton, 2003) |
|  |  | α-pinene, β-pinene, **α-terpineol**, γ-terpinene, 1,8-cineole, borneol, camphen, camphor, **carvacrol**, carvone, *cis*-chrysantenol, *cis*-*p*-mentha2,8-dien-1-ol, *cis*-thujone, citronellal, dihydrocarveol, *cis*-sabinene hydrate, limonene, **linalool**, myrcene, *p*-cimene, sabinene, terpinen-4-ol, **thymol**, *trans*-carveol, *trans*-sabinol, *trans*-thujone, tuj-3-en-10-al, thujanol | (Pellegrini et al., 2017) |
|  | Diterpenes | buddlejone, deoxibuddlejone, hydroxybuddlejone | (Houghton, 2003) |
|  | Bisditerpenes | maytenone | (Houghton, 2003) |
|  | Triterpenes | α-amyrin, β-amyrin, glutinol, lupeol | (Bustamante et al., 2015; Houghton, 2003) |
|  | Sesquiterpenes | buddledin A, buddledin B, buddledin C, buddledone A, buddledone B, dihydrobuddledin A, zerumbone | (Bustamante et al., 2015; Houghton, 2003) |
|  |  | α-humulene, γ-gurjunene, γ-cadinene, δ-cadinene, bulnesol, caryophilla4(12),8(13)-dien5-ol, caryophyllene, elemol, β-cedrene epoxide**, spathulenol,** muurolol, caryophyllene oxide | (Pellegrini et al., 2017) |
|  | Tetraterpenes | crocetin | (Bustamante et al., 2015) |
